# Editorial for the Special Issue “Amorphous and Nanocrystalline Semiconductors: Selected Papers from ICANS 29”

**DOI:** 10.3390/nano13182594

**Published:** 2023-09-20

**Authors:** Kunji Chen, Shunri Oda, Linwei Yu

**Affiliations:** 1 School of Electronic Science and Engineering, National Laboratory of Solid State Microstructures, Nanjing University, Nanjing 210093, China; 2 Quantum Nanoelectronics Research Center, Tokyo Institute of Technology, 2-12-1 Ookayama, Meguro-ku, Tokyo 152-8550, Japan; soda@pe.titech.ac.jp; 3 National Laboratory of Solid State Microstructures, School of Electronics Science and Engineering, Collaborative Innovation Center of Advanced Microstructures, Nanjing University, Nanjing 210093, China; yulinwei@nju.edu.cn

The 29th International Conference on Amorphous and Nanocrystalline Semiconductors served as a continuation of the biennial conference that has been held since 1965. ICANS 29 was held from 23 to 26 August at the campus of Nanjing University—a great venue for global academic researchers, industrial partners, and policy-makers to come together and share their latest progress, breakthroughs, and new ideas on a wide range of topics in the fields of amorphous and nanocrystalline thin films and other nanostructured materials, as well as device applications.

It was the first time that this prestigious event was held in China, and it provided the perfect opportunity for young Chinese researchers and students to more actively participate in academic exchange as a part of the conference and get to know the latest developments in the fields in which they work. And despite a one-year delay due to the COVID-19 pandemic, ICANS 29 still attracted more than 300 paper submissions from 11 countries, including on-site or online oral and poster presentations, which made it truly both a global and hybrid conference.

For this Special Issue on “ICANS29”, twenty-one papers presented at the 29th ICANS have been selected by the Journal of *Nanomaterials* for publication, following a rigorous peer-review process. The scope of this Special Issue is to provide recent developments and research activities in the field of amorphous and nanocrystalline semiconductors. This includes topics such as Si-based, oxide, perovskite, 2D thin films and nanostructures, device applications for TFTs, solar cells, and LEDs, as well as memory devices and emerging flexible electronics and neuromorphic applications. For example, new fabrication technologies of amorphous and nanocrystalline thin films, electronic and optical characteristics, and device applications are presented and discussed in [1,2,3,4,5,6,7,8,9,10,11,12,13,14,15,16,17,18,19,20,21], including theoretical work in an ab initio study [9], controllable growth and formation of Si nanowires [1], nanocrystals [2], and quantum dots [3,4]. There are also several papers that cover emerging memory devices. These include phase change memory [5,6,7,8,9] based on amorphous chalcogenide thin films and memristors based on transition metal oxides acting as an artificial synapse [10,11,12]; the improvement of electro-luminescence (EL) efficiency Er-doped oxide thin films [13,14,15]; 2D semiconductor thin films and perovskite for solar cells and aqueous Zn-air battery [16,17,18,19]; and flexible electronic materials for integrated strain sensors [20,21]. We anticipate that this Special Issue should interest a broad audience in these related fields.

In terms of the content of this Special Issue, the first paper, from Yu’s group [1], details the process of growing a uniform ordered ultrathin Si nanowire (SiNW) array by a nano-stripe-confined approach to produce highly uniform indium catalyst droplets via a relatively new in-plane solid–liquid–solid growth model. The diameters of the ultrathin SiNWs can be scaled down to only 28±4 nm, which opens up a reliable route to batch-fabricate and integrate ultrathin SiNW channels for various high-performance field effect transistors (FET), sensors, and display applications. Xu’s group [2] describes using Si nanocrystals (NCs)/SiC multilayer structures to form uniform SiNCs by plasma-enhanced chemical vapor deposition (PECVD) via the interface-confinement growth approach. The results show that the main EL near 750 nm can be obtained from 4 nm sized NCs. Moreover, it was found that the external quantum efficiency (EQE) of SiNCs/SiC multilayer light-emitting diode (LED) can be improved by phosphor doping. Pi’s group [3] used nonthermal plasma to synthesize Si quantum dots (QDs) hyper-doped with Er at the concentration of ~1% to obtain near-infrared (NIR) light emission at a wavelength of ~830 and ~1540 nm. Furthermore, an ultrahigh η_ET_ (~93%) was obtained owing to the effective energy transfer from SiQDs to Er^3+^. The results suggest that Er-hyper-doped SiQDs have great potential for the fabrication of high-performance near-infrared (NIR) light-emitting devices. Additionally, Miyazaki’s group [4] demonstrated the high-density formation of SiQDs with Ge-core on ultrathin SiO_2_ with the control of highly selective chemical vapor deposition for NIRLED applications. The results show that light emission is attributed to radiative recombination between quantized states in the Ge-core with a deep potential well for holes caused by electron/hole simultaneous injection from the gate and substrate, respectively.

The study of amorphous chalcogenide phase change characteristics and phase change random access memory (PCRAM) devices is one of the most active areas of research featured in this conference. Song’s group [5] investigated the microstructural characterization and electrical properties of the Ta-doped Sb_3_Te_1_ films. The results show that Ta_1.45_Sb_3_Te_1_ has enhanced thermal stability, reduced grain size, and increased the switching speed of PCRAM devices. Wu’s group [6] reported the effect of carbon doping on Sb_2_Te_3_. It was found that the face-centered cubic (FCC) phase of C-doped Sb_2_Te_3_ appeared at 200 °C and began to transform into the hexagonal (HEX) phase at 25 °C. Based on the first principle density functional theory calculation, it was found that the formation energy of the FCC–Sb_2_Te_3_ structure decreases gradually with an increase in C-doping concentration. In addition, Zheng’s group [7] studied the relationship between electron transport and microstructure in mature Ge_2_Sb_2_Te_5_ (GST) alloy. The results indicated that the first resistance dropping in GST films is related to the increase in carrier concentration. However, the second drop is related to the increase in carrier mobility. In order to suppress the crosstalk and provide a high on-current to melt the incorporated phase change materials in the PCRAM array and 3D stacking chips, the authors [8] investigated the influence of Si concentration on the electrical properties of the Si-Te ovonic threshold selector. The results showed that the threshold voltage and leakage current remain basically unchanged with the electrode size scaling down. In addition, Kolobov’s group [9] studied the effect of doping in typical chalcogenide glass As_2_S_3_ with transition metals (TM), such as Mo, W, and V, by using first-principal scaling simulations. The results indicated a strong effect of TM deposits on electronic structures of the a-As_2_S_3_ as well as an appearance of a magnetic response, which suggests that chalcogenide glasses doped with TMs may become a technologically important material. Besides the PCRAM devices, the memristive devices based on a metal–insolate–metal (MIM) structure with a transition metal oxide dielectric layer are yet another kind of emerging memory device discussed in this Special Issue. Ma’s group [10] reported that the controllable memory window of the HfO_2_/TiO_x_ memristive device could be obtained by tuning the thickness ratio of sublayers. As an artificial synapse based on HfO_2_/TiO_x_ memristor, stable, controllable biological functions have been observed, which provides a hardware basis for their integration into the next generation of brain-inspired chips. In addition, they [11] described the a-SiNx:H-film-based resistive switching memory and used the transient current measurements to discover the Si dangling bonds nanopathway in a-SiNx:H resistive switching memory. Moreover, they [12] also employed the capacitance–voltage (C-V) measurements to investigate how to control the carrier injection efficiency in 3D nanocrystalline Si floating gate memory.

Sun’s group [13,14] studied the luminescence characteristics of metal oxides doped by rare-earth elements. Firstly, the amorphous Al_2_O_3_-Y_2_O_3_:Er nanolaminate films prepared by atomic layer deposition with 1530 nm EL were obtained. It was found that the introduction of Y_2_O_3_ into Al_2_O_3_ reduced the electric field for Er excitation, and the EL performance was significantly enhanced. Additionally, a YBO_3_ phosphor co-doped by Bi^3+^ and Gd^3+^ was prepared via high-temperature solid-state synthesis. The strong photon emissions were found under both ultraviolet and visible radiation, which could serve as a potential application for skin treatment. On the other hand, an intriguing phenomenon was discovered by Huang’s group [15]. The PL properties and stability of CsPbBr_3_ QDs films can be enhanced by an a-SiC_x_N_y_:H encapsulation layer prepared using a very-high-frequency PECVD technique. This method not only reduced the impact of air, light, and water on the QDs but also effectively passivated their surface defect states, leading to improved PL efficiency.

There are also two papers on solar cells from Wang’s group [16], which focus on how to employ TiO_2_/SnO_2_ bilayer electron transport layer (ETL) to construct high-performance perovskite solar cells (PSCs). Such a bilayer structure ETL can not only produce a larger grain size of PSCs but also provide a high current density and reduced hysteresis. Their other paper [17] demonstrated high-performance Ag/ITO/CuO_2_/ZnSnN_2_/Au heterojunction solar cells. The crucial technique employed was a nanocrystallization process, which can greatly reduce the electron density of the ZnSnN_2_ sublayer. Song’s group [18] designed 2D van der Waal (vdW) heterostructures, such as BP/BP/MoS_2_, BlueP/BlueP/MoS_2_, BP/graphene/Mos_2_ and BlueP/graphene/MoS_2_, etc., trilayer structures, and stimulated using the first-principles calculation to discover new optoelectronic properties. It is suggested that sophisticated 2D trilayer vdW heterostructures can provide further optimized optoelectronic devices. For the study of 2D materials, Song’s group [19] reported a stable rechargeable aqueous ZN-air battery by using a heterogeneous 2D MoS_2_ cathode catalyst, which demonstrated a capacity of 330mAhg^−^^1^ and a durability of 500 cycles (~180 h) at 0.5 mAcm^−2^. The hydrophilic and heterogeneous MoS_2_ catalysts were prepared through a simple hydrothermal synthesis method.

Finally, this Special Issue features a study of the flexible electronic materials for sensor device applications. Shi and Pan’s group [20,21] presented a scalable porous piezoresistive/capacitive dual-mode sensor with a porous structure in polydimethylsiloxane (PDMS) and with multi-walled carbon nanotubes (MLCNTs) embedded on its internal surface to form a 3D spherical-shell-structured conductive flexible network. This kind of polymer flexible sensor can be assembled into a wearable sensor that has a good ability to detect human motion and can be used for simple gesture and sign language recognition. Moreover, they also proposed a novel approach that combines self-assembled technology to prepare a high-performance flexible capacitive pressure sensor with a microsphere-array gold electrode and a nanofiber nonwoven dielectric material. The results of COMSOL simulations and the experiments showed that the flexible capacitive pressure sensor exhibits excellent performance in pressure measurements and has significant potential for electronic skin applications.

The papers collected here only reflect a portion of the topics presented in this conference. We hope that this Special Issue will stimulate fruitful discussions and cooperation between experts in academia and industry who work in the field of amorphous and nanocrystalline semiconductors. It has also been a wonderful gift to have hosted the ICANS29 in Nanjing, China.

We would like to thank all of the authors and the *Nanomaterials* Editorial Office for their great contributors and excellent work.
